# Non-LTR R2 Element Evolutionary Patterns: Phylogenetic Incongruences, Rapid Radiation and the Maintenance of Multiple Lineages

**DOI:** 10.1371/journal.pone.0057076

**Published:** 2013-02-25

**Authors:** Andrea Luchetti, Barbara Mantovani

**Affiliations:** Dipartimento di Scienze Biologiche, Geologiche e Ambientali, Università di Bologna, Bologna, Italy; University of Muenster, Germany

## Abstract

Retrotransposons of the R2 superclade specifically insert within the 28S ribosomal gene. They have been isolated from a variety of metazoan genomes and were found vertically inherited even if their phylogeny does not always agree with that of the host species. This was explained with the diversification/extinction of paralogous lineages, being proved the absence of horizontal transfer. We here analyze the widest available collection of R2 sequences, either newly isolated from recently sequenced genomes or drawn from public databases, in a phylogenetic framework. Results are congruent with previous analyses, but new important issues emerge. First, the N-terminal end of the R2-B clade protein, so far unknown, presents a new zinc fingers configuration. Second, the phylogenetic pattern is consistent with an ancient, rapid radiation of R2 lineages: being the estimated time of R2 origin (850–600 Million years ago) placed just before the metazoan Cambrian explosion, the wide element diversity and the incongruence with the host phylogeny could be attributable to the sudden expansion of available niches represented by host’s 28S ribosomal genes. Finally, we detect instances of coexisting multiple R2 lineages showing a non-random phylogenetic pattern, strongly similar to that of the “library” model known for tandem repeats: a collection of R2s were present in the ancestral genome and then differentially activated/repressed in the derived species. Models for activation/repression as well as mechanisms for sequence maintenance are also discussed within this framework.

## Introduction

R2 is one of the most studied retrotransposons among the non-LTR R elements specifically inserting into the 28S (R1, R2, R4, R5, R6, R9, and RT families) or into the 18S (R7 and R8 families) ribosomal genes ([1], [2] and references therein). R2 elements have a single open reading frame (ORF) flanked by two untranslated sequences of variable length. Conserved regions of the ORF are the central reverse transcriptase (RT) domain, the DNA-binding motifs [3] at the N-terminus and the endonuclease domain at the C-terminus. The C-terminal end of the R2 protein includes a cysteine-histidine (zinc finger) motif (CCHC [4]), while the N-terminal domain can contain one (CCHH), two (CCHH; CCHH), or three (CCHH; CCHC; CCHH) zinc finger motifs [5].

R2 inserts into the specific sequence 5′-TTAAGG↓TAGCCA-3′ of the 28S rRNA gene through a target primed reverse transcription mechanism [6]. R2’s RT shares more similarity with the lentiviral RTs, like the HIV-1 one, being as well able to synthesize DNA at low dNTP concentrations with high error rate. Moreover, R2 was suggested to retrotranspose also in non-dividing cells, but its long-term nucleotide substitution rate does not appear significantly higher than that associated with cellular DNA replication [7]. During R2 reverse transcription, first strand synthesis may be incomplete, so that a 5′ end truncated copy is inserted; length variations at the R2 5′ end can be used to track element activity [8].

R2 is one of the “early branching” elements among non-LTR retrotransposons ([9] and references therein). R2 occurs in the four triploblastic phyla Platyhelminthes, Arthropoda, Echinodermata, and Chordata, and in the diploblastic phylum Cnidaria. Its origin is assumed to date back before the Radiata - Bilateria cladogenesis, being therefore a very ancient resident of metazoan genomes [1]. The specificity of insertion within a repetitive sequence family may have played a major role for its long-term survival in the host genome [10].

The interaction between the R2 transposition/insertion and the rDNA evolutionary dynamics mirrors a complex interplay. For example, R2 insertions often produce the deletion of upstream rDNA segments (transposition-mediated deletions), which could delete either inserted or uninserted 28S rRNA copies; on the other hand, the non-Mendelian genomic turnover mechanisms (GTM) of the rDNA repeats may either eliminate inserted 28S rDNAs or may replace the lost ones with new uninserted copies, thus providing new sites for further R2 insertions [11], [12], [13]. Therefore, R2 dynamics is somehow involved in the molecular drive of the ribosomal locus, i.e. the dual process comprising *i*) the intragenomic homogenization of sequence variants, through GTM such as unequal crossing-over, gene conversion and rolling circle replication, and *ii*) the sequence variant fixation within a group of reproductively linked bisexual organisms [14], [15]. Molecular drive leads to the pattern of concerted evolution observed for tandem repetitive sequences: the sequence divergence between repeats within the same evolutionary unit (a species, a subspecies or a population) is significantly lower than between repeats sampled from different evolutionary units of the same taxonomic rank.

R2 phylogeny computed on the C-terminal half of the RT domain shows the presence of 11 sub-clades clustering in four main clades. These are consistent with the number of zinc finger motifs (ZF) at the N-terminal end: the A clade groups elements with three ZF motifs (CCHH, CCHC, CCHH), while the D clade has only one ZF (CCHH) corresponding to the third one of clade A; the C clade lacks the second zinc finger of clade A and the amino-terminal structure of the R2-B clade has not been determined yet [5], [16], [17].

On the other hand, the tree topology does not mirror the host phylogeny: elements from distant species can be found in the same sub-clade, while elements from related species lie in different (sub-)clades. Only in few instances a pattern of strict vertical inheritance occurs: it happens for the insect genera *Drosophila* and *Reticulitermes*, the Ixodida ticks and the crustacean genus *Lepidurus* [18], [19], [20], [21]. As a general remark, though, it is to be pointed out that, even if the main topology can be recognized, trees are weakly supported in most instances [1], [2], [13], [16], [20], [21].

To further complicate the scenario, some species, such as *Lepidurus couesii, Tenebrio molitor, Popilia japonica, Nasonia vitripennis, Ciona intestinalis* and *Mauremys reevesii*, host multiple R2 lineages [17], [21], [22], [23].

Inconsistencies between R2 and hosts phylogenies have been explained through the coexistence in a genome of paralogous R2 elements, followed by one or more lineages extinction ([9] and reference therein). Furthermore, the coexistence of multiple R2 lineages has been explained either by genomic control or by host population dynamics; however, both models have been only suggested but not directly based on actual data.

We here present a database survey that led to the identification of 21 new R2 elements; the new data have been, then, compared with all already available R2 sequences and analyzed in a thorough phylogenetic framework with the aim to gain a clearer evolutionary scenario. First of all, the new data specifically highlights the ZF structure of the B-clade. Phylogeny, likelihood mapping analysis and branch length distribution on all available elements show a pattern consistent with a R2 rapid radiation, possibly overlapping with that of metazoan; this may have contributed to the observed lack of congruence of the R2/host phylogenies. Moreover, based on the observed phylogenetic distribution of coexisting R2 lineages among related taxa, we propose a model that may explain the maintenance of multiple R2 lineages starting from R2 collections in ancestral genomes.

## Materials and Methods

### Databases Search for R2 Sequences Isolation

The databases NCBI (http://www.ncbi.nlm.nih.gov/), Human Genome Sequencing Center (Baylor College of Medicine, https://www.hgsc.bcm.edu/content/genome-data), Bogas (http://bioinformatics.psb.ugent.be/orcae/overview/Tetur) and SmedGD v. 1.3.14 (http://smedgd.neuro.utah.edu/index.html) were scanned through BLAST (*tblastn* algorithm), with different R2 RT amino acid sequences as probes: R2Bm (*Bombyx mori*), R2Tc (*Triops cancriformis*), R2SmA (*Schistosoma mansoni*), R2Amel (*Apis mellifera*) [5], [13]. BLASTing a 120 bp 28S sequence centred on the target insertion site of R2 further validated positive hits, being this well conserved region able to match homologous sequences across metazoan. These new R2 sequences are available on the Authors’ website (http://www.mozoolab.net/index.php/downloads.html; Table 1). Further R2 sequences, never analyzed before, were retrieved in the Repbase Update collection (http://www.girinst.org/repbase/index.html [24]; Table 1). R2 nucleotide sequences with identity values >90% were considered as belonging to the same family.

**Table 1:** List of newly analyzed R2 sequences.

Phylum		*Subphylum*/Class(Order)	Species	Acronyms	Source	R2 length (bp)	ORF length (bp)
Platyhelmintes							
	Turbellaria		*Schmidtea mediterranea* A	R2Smd-A	Repbase	5417	3096
			*S. mediterranea* B	R2Smd-B	genome sequence	4004	3216
	Trematoda		*Schistosoma japonicum*	R2Sj	genome sequence	4275	3774
Nematoda							
	Adenophorea		*Trichinella spiralis*	R2Ts	Repbase	3690	3255
Arthropoda							
	*Chelicerata*						
	Arachnida		*Tetranychus urticae*	R2Tu	genome sequence	3894	3261
						
	*Pancrustacea*Insecta						
	(Hemiptera)		*Acyrthosiphon pisum*	R2Ap	genome sequence	3447	3162
						
	(Coleoptera)		*Tribolium castaneum* A	R2Tcs-A	genome sequence	4171	3828
			*T. castaneum* B	R2Tcs-B	genome sequence	4517	3708
			*T. castaneum* C	R2Tcs-C	genome sequence	3348	3093
	(Hymenoptera)		*Linepithema humile*	R2Lh	genome sequence	4110	3030
			*Camponotus floridanus*	R2Cf	genome sequence	3472	3147
			*Solenopsis invicta*	R2Si	genome sequence	3410	3162
			*Pogonomyrmex barbatus* A	R2Pb-A	genome sequence	4504	3339
			*P. barbatus* B	R2Pb-B	genome sequence	3543	3258
			*Harpegnathos saltator*	R2Hs	genome sequence	4345	3765
			*Bombus terrestris*	R2Bt	genome sequence	3540	3267
			*Bombus impatiens*	R2Bi	genome sequence	3748	3405
			*Megachile rotundata*	R2Mrt	genome sequence	3572	3381
Chordata							
	*Vertebrata*						
	Cephalaspida		*Petromyzon marinus* A	R2Pm-A	Repbase	5038	3255
			*P. marinus* B	R2Pm-B	Repbase	2988	2739
	Aves		*Taeniopygia guttata*	R2Tg	Repbase	4673	4170

Presently identified R2s were then gathered in a single dataset with all elements available from literature (Eickbush lab, http://blogs.rochester.edu/EickbushLab/?page_id=602; [5], [13], [19], [20], [21]; Table S1). R8 and R9 elements, closely related to R2 [1], [2], have been also included in phylogenetic analyses.

Each element is indicated by the host species acronym followed by a number if isolated from different individuals (i.e. different genomes) or by a capital letter if they have been isolated from a single individual (the same genome).

### R2 Sequence Analysis and Phylogenetics

In order to maintain data congruence with previous analyses, the C-terminal ends of the R2 proteins (including the RT domain) were considered. Global sequence alignment has been obtained through MAFFT 6.8 software [25], using L-INS-i parameters. Alignment was then refined with Noisy 1.5 [26] in order to remove potentially homoplasious sites and alignment errors giving wrong phylogenetic signals. Before phylogenetic analyses, the strength of the phylogenetic signal was tested by mapping the likelihood of 5000 quartets [27] sampled either randomly or from the four clusters defined on the basis of the zinc finger pattern. The algorithm used is implemented in the Tree-Puzzle 5.2 package [28]. Pairwise genetic divergence (p-distance), the choice of the best amino acid substitution model for phylogeny (rtREV+G) and the Minimum Evolution tree (ME), with 1000 bootstrap replicates, were calculated with MEGA 5.05 [29]. A Maximum Likelihood tree (ML), with 100 bootstrap replicates, was obtained with PhyML [30], implemented on DIVEIN server [31]. Bayesian Inference (BI) was carried out with MrBayes 3.2 [32]: the Markov Chain Monte Carlo process was set on two simultaneous tree searches running for 2×10^6^ generations, with trees sampled every 500 generations, for a total of 4001 trees. The burnin was set to 10%.

## Results

### R2 Sequences Characterization

Sixteen R2 elements were identified from available genomes: 13 from insects, one from the spider mite and two from flatworms. Moreover, five R2 sequences were retrieved in Repbase Update: three from Chordata, one from Nematoda and one from flatworms. In four taxa multiple R2 lineages were detected, coexisting the same genome: three families in the flour weevil *Tribolium castaneum* and two families each in the harvester ant *Pogonomyrmex barbatus*, in the planarian *Schmidtea mediterranea* and in the sea lamprey *Petromyzon marinus*.

All R2s were full-length elements but two sequences: the *P. marinus* B element (R2Pm-B, drawn from the Repbase Update) and the jumping ant *Harpegnathos saltator* element (R2Hs, isolated from the genome sequence). For this latter element it was not possible to determine the 5′ UTR and the sequence was considered from the beginning of the ORF.

The identified elements insert specifically in the expected 28S site (5′-AAGG↓TAGC-3′): this region is well conserved across all newly analyzed species but *T. castaneum*, showing a point mutation immediately upstream the insertion site (Figure S1).

Full-length, newly isolated elements fit to the canonical R2 structure, with length ranging from 3348 bp to 5417 bp and showing a single ORF from 3030 bp to 4170 bp long (Table 1).

**Figure 1:**
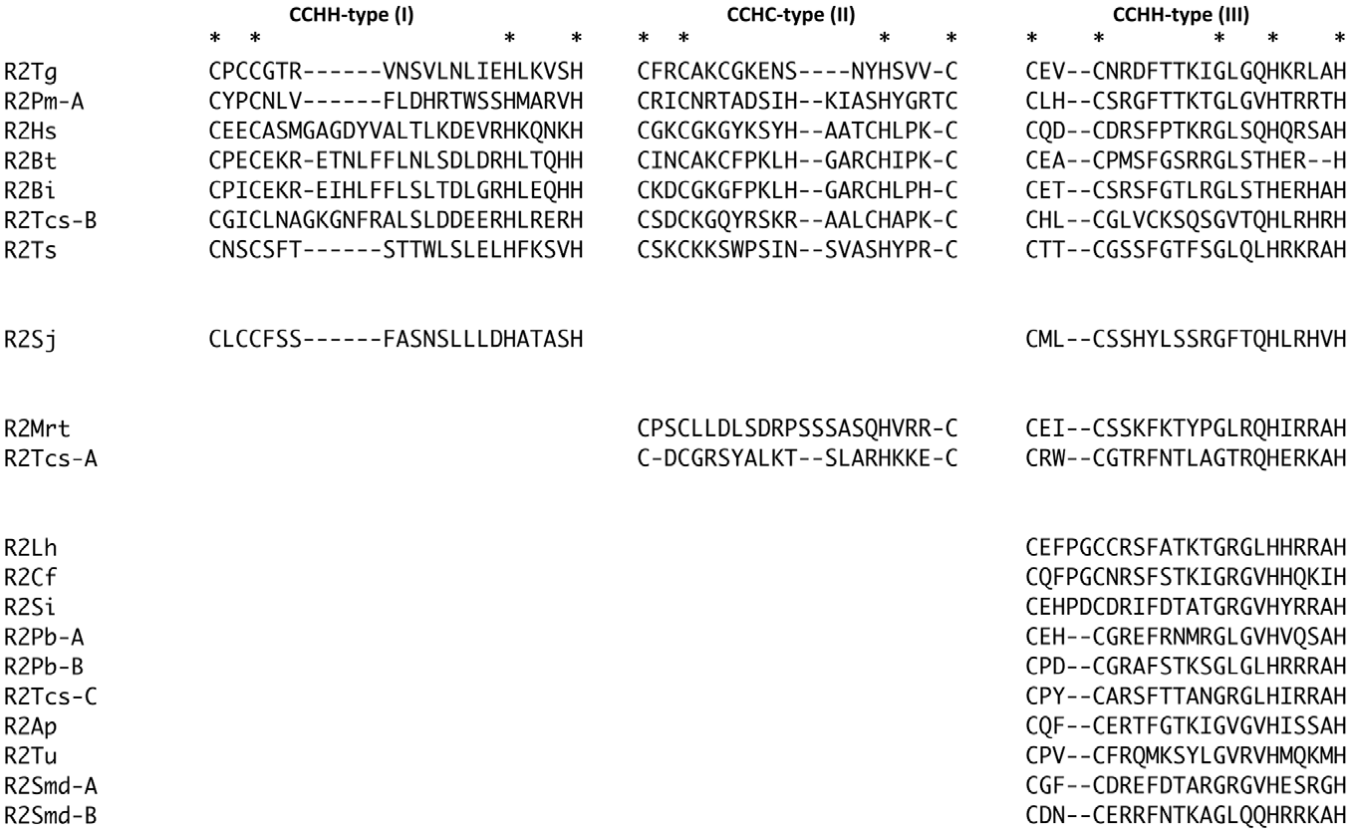
N-terminal zinc finger motif patterns of the presently analyzed R2 protein sequence. Asterisks mark C and H residues characterizing the zinc finger motif; acronyms are as in Table 1.

The N-terminal end of the analyzed R2 proteins presents one (10 elements), two (three elements) or three (seven elements) ZF motifs (Figure 1). Only one ZF domain was identified for R2Pm-B, due to the incompleteness of the ORF region. It is also to be noted that the three R2s with two ZFs do not have the same pattern: while *S*. *japonicum* R2 has the already known ZF domains I+III, like the elements from the congeneric *S. mansoni*, the *T. castaneum* A and the *M. rotundata* elements have ZF II+III (Fig. 1).

### Protein Alignment and Phylogenetic Analyses

In order to exclude from the final dataset amino acid positions with missing data, the alignment was performed on the 3′ half of the R2 protein that is available for all the already sequenced elements. This region encompasses the 3′ half of the RT domain, including part of the more conserved finger/palm sub-domain and the more variable thumb sub-domain, and the C-terminal end embodying a CCHC-type ZF and the RLE domain. The length of the final alignment, which includes 87 OTUs (operational taxonomic units, i.e. R2 protein sequences), was equal to 834 amino acid positions; the elimination of potentially homoplasious sites reduced the length to 542 positions, the software deleting mainly blocks with indels.

**Figure 2:**
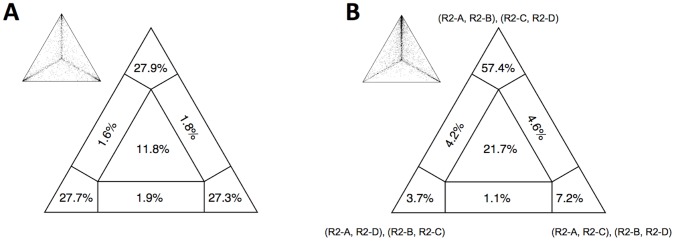
Likelihood quartets mapping. A) Distribution of randomly sampled quartets; B) distribution of quartets with sampling forced from each of the four clusters defined by the zinc finger patterns (R2-A, R2-B, R2-C and R2-D). Areas at triangle’s angles are those with resolved topology, the others are with conflicting/unresolved topologies. In B) at each angle is indicated the corresponding topology. Small triangles represent detailed distributions of quartet topologies, while big triangles summarize the percentages of topologies falling in each area.

Likelihood mapping was then used to assess the reliability of the phylogenetic signal of the refined alignment. The analysis was run twice: in the first run, the algorithm sampled quartets randomly, while in the second run OTU choice was forced for quartets from the four clusters expected on the basis of the number of ZF motifs (R2-A, R2-B, R2-C and R2-D). Data from the two analyses were significantly different (χ^2^ = 758.6, d.f. = 2, *P*<0.001): in the random quartet choice, the resolved topologies are the 82.9%, the conflicting ones the 5.3% and the unresolved 11.8%, while - when forcing for the four clusters - values are 68.3%, 9.9% and 21.7%, respectively (Figure 2). It should be noted that in the latter case, the 57.4% of the quartets fall within the area of the resolved topology ((R2-A, R2-B),(R2-C, R2-D)) (Figure 2B). These results indicate that a topology quite similar to that expected from previous analyses is the most supported, even if with a higher fraction of conflicting/unresolved topologies.

**Figure 3:**
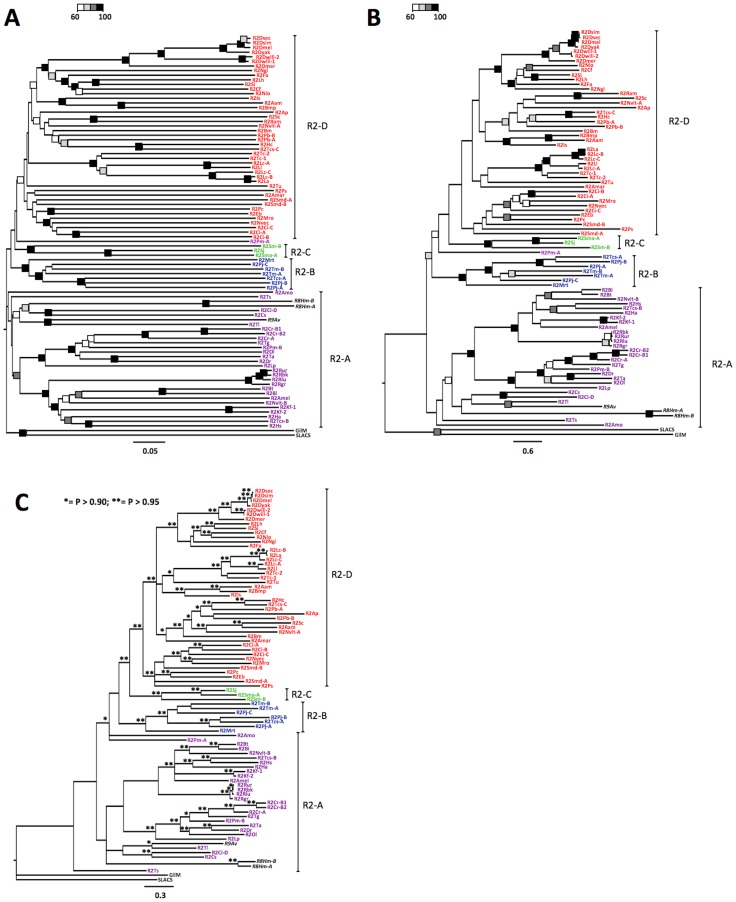
Phylogenetic analyses on all available R2 amino acid sequences. A) Minimum evolution (ME; SBL = 23.62); B) Maximum Likelihood (ML; -lnL = 68687.39); C) Bayesian Inference (BI; -lnL = 70547.77). The four main clades (R2-A, R2-B, R2-C and R2-D) are indicated in each tree. In the ME and ML trees, grey-scale squares indicate bootstrap value intervals as represented in the schematic legend within A) and B) panels’ top left corner, respectively. In the BI tree, asterisks indicate posterior probability *P* values as in the C) panel’s top left corner legend. Acronyms are as in Table 1 and Table S1.

The phylogenies based on ME, ML and BI analyses are largely congruent but nodal supports vary widely among trees: the ML tree is the most unresolved, while the BI shows generally higher nodal supports (Figure 3). The four clades expected on the basis of previous analyses are recognizable in each phylogenetic reconstruction but their monophyly is not always supported: R2-B and R2-C clades are significantly supported in all analyses, the R2-D clade is well defined only in the BI analysis and the R2-A clade constitutes a basal polytomy in all analyses. R8 and R9 elements, according to previous analyses [1], [2] and to the number of zinc finger motifs (I+II+III), are placed in the R2-A clade.

The newly identified R2 sequences distribute following the number of zinc fingers (3 ZFs –clade A; 2 ZFs, motifs I+III – clade C; 1 ZF –clade D; Fig. 1 and 3). *M. rotundata* and *T. castaneum* A elements, showing the new ZF pattern made by motifs II+III, fall within the clade B whose ZF number and pattern were not characterized so far.

The only exception to the ZF-based clustering is represented by the *P. marinus* R2Pm-A element whose position is unresolved in the ME tree (Figure 3A), or it is placed basal to the (R2-C, R2-D) group in the ML tree (but with low bootstrap value; Figure 3B), or it is basal to the (R2-B, R2-C, R2-D) cluster in the BI elaboration, together with R2Amo (with significant posterior probability; Figure 3C).

Within phylogenetic trees, variability does not appear to be evenly distributed over time: indeed terminal branch lengths are always quite long. In order to test this observation, terminal branch lengths (i.e. from the OTU to the nearest node) were compared with internodal lengths (i.e. from internal node to the nearest internal node) for all phylogenetic elaborations: terminal branch lengths are always significantly longer than internodal branch lengths (Table 2).

**Table 2:** *t*-test for comparison between terminal and internodal branch lengths in the Minimum Evolution (ME), Maximum Likelihood (ML) and Bayesian Inference (BI) trees.

Tree	Avg. terminal branch length	Avg. internodal branch length	*t* (d.f.)	*P*
ME	0.225	0.042	12.57 (126.1)	*<0.001*
ML	0.486	0.225	5.23 (148.9)	*<0.001*
BI	0.420	0.191	6.04 (145.1)	*<0.001*

**Figure 4:**
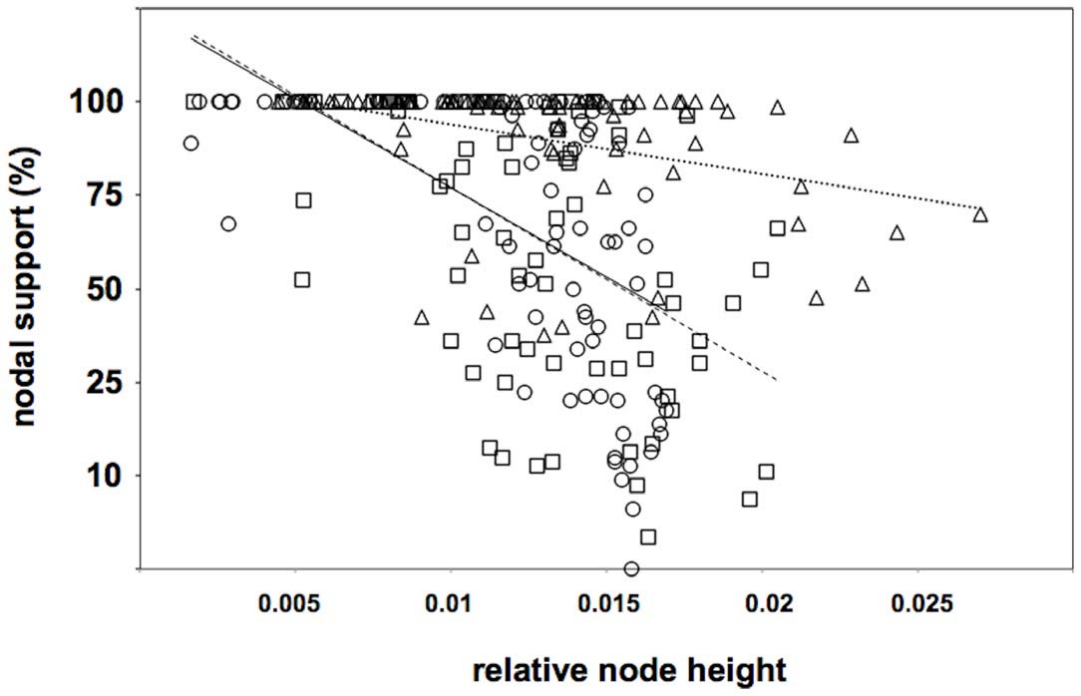
Scatter plot with correlation between relative node height and nodal support for the three phylogenetic analyses. Circles: ME; squares: ML; triangles: BI. Solid line indicates correlation for ME (Pearson r = −0.554; *P*<0.001), dashed line for ML (Pearson r = −0.548; *P*<0.001) and dotted line for BI (Pearson r = −0.356; *P*<0.001). Relative node height = node height/sum of branch length. Nodal supports are either bootstraps (ME, ML) or posterior probabilities (BI) expressed in percentage.

Moreover, also nodal supports appear to have a biased distribution within trees, the lower values being observed at deepest nodes. Relative node heights were, therefore, correlated with the corresponding nodal support values (either bootstrap or posterior probability): in all elaborations (ME, ML, and BI) there is a statistically negative correlation (i.e. the deeper the node, the lower the nodal support; Figure 4).

As a general remark, R2 phylogeny does not overlap that of the corresponding hosts but for some instance of local congruence, in line with previous analyses: *i*) the *Drosophila* spp. clade [18], *ii*) the European Notostraca clade (genera *Triops* and *Lepidurus*) [21], *iii*) the hard ticks clade [20] and *iv*) the *Reticulitermes* termites clade [19].

As far as multiple coexisting R2 lineages are concerned, the distribution and phylogenetic analyses demonstrate quite clearly that - even when falling in the same clade - they never cluster together, the only exception being *Mauremys reevesi* A, B1 and B2 elements (clade A) and *Ciona intestinalis* A and B elements (clade D) (Table 3, Figure 3).

**Table 3:** Clade distribution of R2 lineages co-occurring in the same genome.

Species	R2 lineage	R2 clade			
		A	B	C	D
*Schmidtea mediterranea*	A				+
	B				+
*Schistosoma mansoni*	A			+	
	B			+	
*Lepidurus couesii*	A				+
	B				+
	C				+
*Tenebrio molitor*	A		+		
	B		+		
*Tribolium castaneum*	A		+		
	B	+			
	C				+
*Nasonia vitripennis*	A				+
	B	+			
*Popilia japonica*	A		+		
	B		+		
	C		+		
*Pogonomyrmex barbatus*	A				+
	B				+
*Ciona intestinalis*	A				+
	B				+
	C				+
	D	+			
*Petromyzon marinus*	A	?	?	?	?
	B	+			
*Mauremys reevesii*	A	+			
	B1	+			
	B2	+			

**Figure 5:**
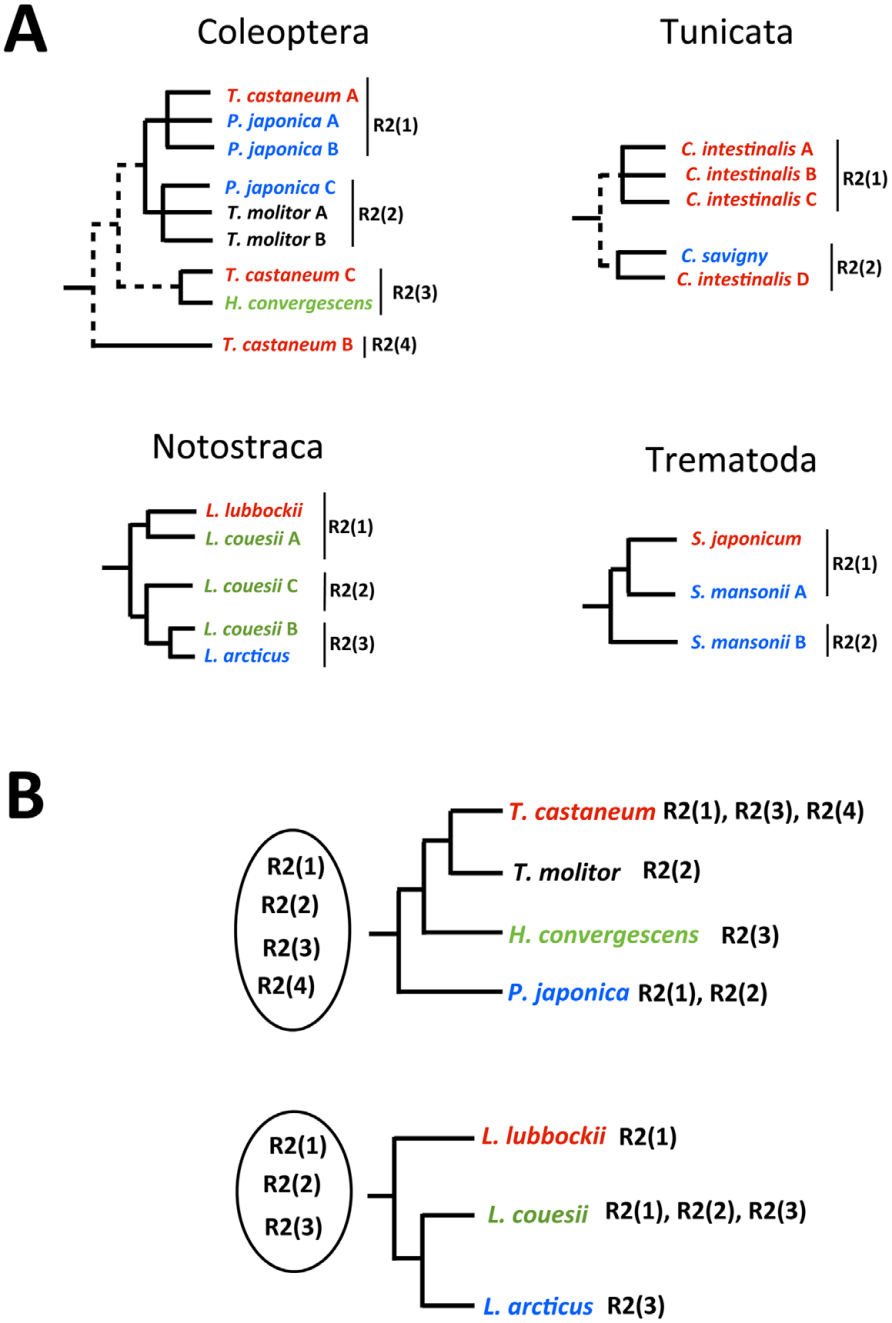
Summary of phylogenetic relationships between multiple coexisting R2 lineages with elements from phylogenetically related species. A) Schematic drawing of relationships as emerged from phylogenetic analyses. Dashed lines indicate relationships across the main clades (R2-A, R2-B, R2-C and R2-D). B) Mapping of R2 lineages distribution on coleopteran and notostracan phylogenies.

On the other hand, as summarized in Figure 5a, multiple R2 lineages – either pertaining to the same clade or to different ones - consistently cluster with elements identified in closely related species in four instances: *i*) two out of three R2 lineages from *T. castaneum* group together with other coleopteran elements and the same can be observed for those from *T. molitor* and *P. japonica*; *ii*) in Tunicata, the only divergent *C. intestinalis* linage (D, falling within the R2-A clade) cluster together with the congeneric *C. savignyi*; *iii*) of the three notostracan *L. couesii* elements, one groups with *L. lubbockii* and one with *L. arcticus*; *iv*) the *S. mansoni* A element is more similar to the *S. japonicum* R2 than to the *S. mansoni* B.

## Discussion

The non-LTR retrotransposon R2 is one of the most studied mobile elements, because of its wide occurrence across the animal Kingdom and its peculiar insertion target, the 28S rRNA gene. Nevertheless, some aspects of its biology and evolution are still unknown. Here we present the analysis of 16 new elements, together with five R2s deposited in the Repbase Update database but never included in a wider, specific study.

All newly identified elements insert within the known 28S rDNA target site that was found conserved across all analyzed genomes. The only exception is the *T. castaneum* 28S target site, showing an A→T transvertion two positions upstream the first cleavage site. This point mutation has been observed also in a number of other metazoan lineages such as ticks, mites, insects and nematomorphan worms [20] but it does not appear to have compromised the R2 target recognition.

Full-length R2s show the typical structure with a single ORF encoding a protein with 1–3 ZF motifs at the N-terminal end, a RT domain and a CCHC-type ZF plus a RLE endonuclease domain at the C-terminal end. The ZF pattern at the N-terminal end is known to have a discriminant power between R2 major clades [5]. The R2-A clade shows three ZF motifs and, branching first in the phylogeny, it has been suggested to represent the ancestral state. Only the *P. marinus* A element shows an unclear position within phylogenies: it has three ZFs, but it is placed outside the R2-A basal group. While nodal supports would not lead to conclusive hypothesis (see below), this R2 is highly divergent from the other three-ZF elements. It could be the product of a recombination event between two R2s coexisting within the *P. marinus* genome, maybe one similar to R2Pm-B (having three ZFs). This specific issue needs, however, deeper analyses to draw conclusive explanations.

On the other side, the R2-D clade appears as the most derived and shows just a single ZF corresponding to the CCHH-type motif III. R2-C elements have ZF motifs I+III and the only representative exhibiting this pattern here characterized from *S. japonicum* was correctly placed in this clade. Two further elements, one from *T. castaneum* (element A) and the other from *M. rotundata*, showed a previously unknown ZF pattern: indeed, they have the two ZF motifs II and III. In the phylogenetic analyses they are unequivocally placed within R2-B clade that had, until now, an undetermined ZF pattern due to sequence incompleteness of the previously isolated elements: therefore, elements of the clade R2-B show a new ZF domain configuration.

Beside the indication of R2-A as the ancestral clade, it is difficult to trace the ZF evolutionary trajectory on obtained phylogenetic trees because of a generally low statistical support to deeper nodes. This problem already occurred in a number of previous analyses carried out on more restricted sequence samplings [1], [2], [13], [16], [20]. Likelihood mapping analysis indicated that a significant fraction (28.6%) of the possible quartets representing the four ZF-based clades are, actually, unresolved or with conflicting topologies; moreover, deeper nodes are averagely less supported than the more derived ones. These evidences are consistent with a general low stability of nodes representing earlier cladogenetic events. In addition, the peculiar branch length distribution within the obtained trees is to be considered: the comparison between terminal and internodal branch lengths indicates that the latter are significantly shorter.

Short internodal branch lengths and poor nodal supports can be due to two main causes: *i*) a low-resolution power of the characters used or *ii*) past rapid radiation events, i.e. deep cladogenetic events occurring in a relatively short time span [33]. When comparing retrotransposons, the RT domain is considered the best character set to use, being well conserved across element lineages [34]. The presently analyzed dataset include both conserved and variable regions of the RT domain and this has been already shown to increase the accuracy of the phylogenetic reconstruction [35]. Moreover, the alignment was cleaned from potentially homoplasious amino acid positions, possibly deriving from parallel/back mutations or constituting ambiguous/unalignable regions, which could disturb the phylogenetic signal. Finally, in the likelihood mapping, our dataset proved to give resolved quartets in the 71.4% of the samplings and the 61.7% concentrate in the area of the topology similar to that expected. Therefore, we can consider our dataset as well performing and the alternative explanation of a past rapid radiation appears as the most likely.

R2 elements belong to an early-branching superclade of non-LTR retrotransposon, being already present at the splitting between Radiata and Bilateria [1], [10]. However, dating the origin of this element is difficult, also because its phylogeny does not always follow the host one. Divergence *vs* age evaluations, besides revealing the absence of horizontal transfer, placed the origin of R2 somewhere between 600 and 850 Myr ago [5], [10], this approximate dating corresponding to the Pre-Cambrian Era. Shortly after 600 Myr ago started a huge and relatively rapid eumetazoan expansion/diversification, during the Ediacaran/Early Cambrian era (the so-called “Cambrian explosion”): within this time span, animals occupied and generated new ecological niches [36]. The close temporal proximity of the eumetazoan diversity explosion and the inferred R2 rapid radiation could, then, indicate a possible causal link: as animals radiated, they provided also available genomic niches - i.e. 28S rDNAs - for R2 demographic expansion.

Being ruled out the possibility of R2 elements’ horizontal transfer, the observed phylogenetic pattern has been explained with a high diversification/extinction rate of paralogous lineages, together with instances of strictly vertical inheritance [5], [16], [18], [19], [20], [21]. Incomplete lineage sorting due to fast host speciation may have further hindered the correct phylogenetic signal: therefore, the possible overlapping between metazoan and R2 radiations may contribute to explain the lack of congruence between the two phylogenies.

Present analyses, carried out on the larger R2 dataset analyzed so far, show the expected pattern of host/R2 phylogeny: a general inconsistency with occasional local congruence. On the other hand, here, a new clustering pattern emerges involving the elements coexisting in the same genome (Table 3 and Figure 5): the close relationships between R2s from phylogenetically related genomes that also harbour other diverging element lineages constitute a peculiar issue.

The origin and the presence of multiple rDNA-targeting retrotransposon lineages (evidenced also by the late-branching R1 retrotransposons superclade) is still a matter of debate, since competition for insertion sites and host fitness would limit the expansion of active elements. The origin of multiple lineages targeting the same 28S site has been explained by either independent copy number regulation or host species population properties, such as size, distribution and structuring [22], [23], [37]. In the former case, every single element lineage may expand within the rDNA locus when its occurrence approaches very few copies; in the latter instance, if the host species distribution range is fragmented, elements of a given lineage can be lost and replaced by a new one in isolated populations and later reintroduced by occasional gene flow. Under this “isolated population” model, possibly coupled with high diversification/extinction rates of paralogous lineages, a substantial diversification of R1/R2 families as well as the presence of multiple lineages can be expected. However, this may not entirely explain the pattern observed here. For example, in both *L. couesii* and *T. castaneum*, two out of three elements cluster with elements from related species; in Coleoptera this happens even in different R2 clades (R2-B and R2-D): these similarities are more likely the result of their origin from a common ancestral pool of sequences than due to the parallel evolution of newly arisen lineages.

Kojima and Fujiwara [5] suggested that some R2 sub-clades are made by paralogous lineages implying that multiple, coexisting lineages have been maintained for a while. Thus, the observed evolutionary pattern can be explained by *i*) the coexistence of a collection of R2 lineages inherited from a common ancestral genome and *ii*) the selection/expansion of some lineages when conditions are favourable (reconstructed, as an example, for Coleoptera and Notostraca in Figure 5b). Interestingly, this scenario parallels the “library” hypothesis, first formulated to explain the presence of multiple families of (peri)centromeric satellite DNAs in the same genome but applicable to tandem repeat families in general (reviewed in [38]): starting from a collection (the library) of different tandem repeat families within an ancestral genome, selective pressures and molecular drive (able to multiply and/or delete copies of a given family) can lead to the expansion of a single repeat family becoming the predominant in a given derived genome, the other families being maintained at low copy number or even lost. Therefore, when screening derived genomes, just one family or more families shared by different species can be detected [39], [40], [41]. While this peculiar pattern was only partly evident from the Kojima and Fujiwara analysis [5], in this paper we provide the first unequivocal evidence of this same pattern in Coleoptera and in Notostraca taxa (Figure 5b). Obviously, non-LTR elements are not tandem repeats but they actually “live” within a tandem-repeats array, the rDNA. A co-evolutionary dynamics between R2 and rDNA has been already assessed, suggesting that rDNA-specific elements may be subjects to mechanisms ruling the evolution of their niche (see for example [12], [13]). Therefore, it can be hypothesized that, given a library of rDNA-specific retroelements within an ancestral genome, they can be inherited by the derived population/species but only one (or few) lineage(s) can be active and can reproduce while the others are silenced, their number progressively decreasing.

Eickbush and co-workers demonstrated that active R1/R2 copies are evenly distributed across the rDNA array; on the contrary, elements can be epigenetically silenced when clustered in a restricted region of the array [42]. Active lineages can be silenced, or silenced lineages can be re-activated, when the rDNA undergoes to structural changes because of molecular drive mechanisms leading to concerted evolution. In this scenario, an active lineage is silenced and/or it is reduced in copy number and another one still residing within that genome can be unleashed, replacing the former as the predominant.

It is interesting to consider what could be the fate of silenced sequences: indeed, these copies may accumulate mutations that could interfere with their ability to be mobilized, for example, disrupting the ORF. What does prevent the degeneration of silenced R1/R2 lineages? In one of the early work on R1/R2, it has been suggested that, together with the rDNA unit, element sequences may undergo concerted evolution, thus maintaining the sequence identity throughout generations [43]. Therefore, it is possible that silenced copies maintain their integrity through the same homogenization mechanisms that guarantee the concerted evolution of rDNA.

Obviously, as for the species-specific amplification of divergent tandem repeat families described by the library hypothesis, the active lineage replacement is expected to occur without any correlation with the host phylogeny: this may give a clue on why local congruencies between element and host phylogenies are only occasionally observed [5].

As a general remark, it is to be noted that a pattern similar to that evidenced here has been observed for the randomly inserting DINE-1 non-autonomous element in *Drosophila* species: although the library model is not specifically mentioned, it has been explained with a quite similar mechanisms [44]. Future studies specifically addressing this issue would certainly clear the evolutionary scenario of multiple transposable elements lineages coexisting in the same genome.

## Supporting Information

Figure S1 Insertion sites within the host species 28S rRNA of newly identifies R2 elements. Alignment of the 28S’s 20 bp upstream and 20 bp downstream the R2 insertion site.
(DOC)

Table S1 List of previously identified R2 sequences used for the phylogenetic analysis. Host species names, acronyms and references for each R2 element.
(DOC)
