# Supplementary figures and images for: Non-LTR R2 Element Evolutionary Patterns: Phylogenetic Incongruences, Rapid Radiation and the Maintenance of Multiple Lineages
Source: PLoS One. 2013 Feb 25;8(2):e57076. doi: 10.1371/journal.pone.0057076 (PMC3581529; doi:10.1371/journal.pone.0057076)

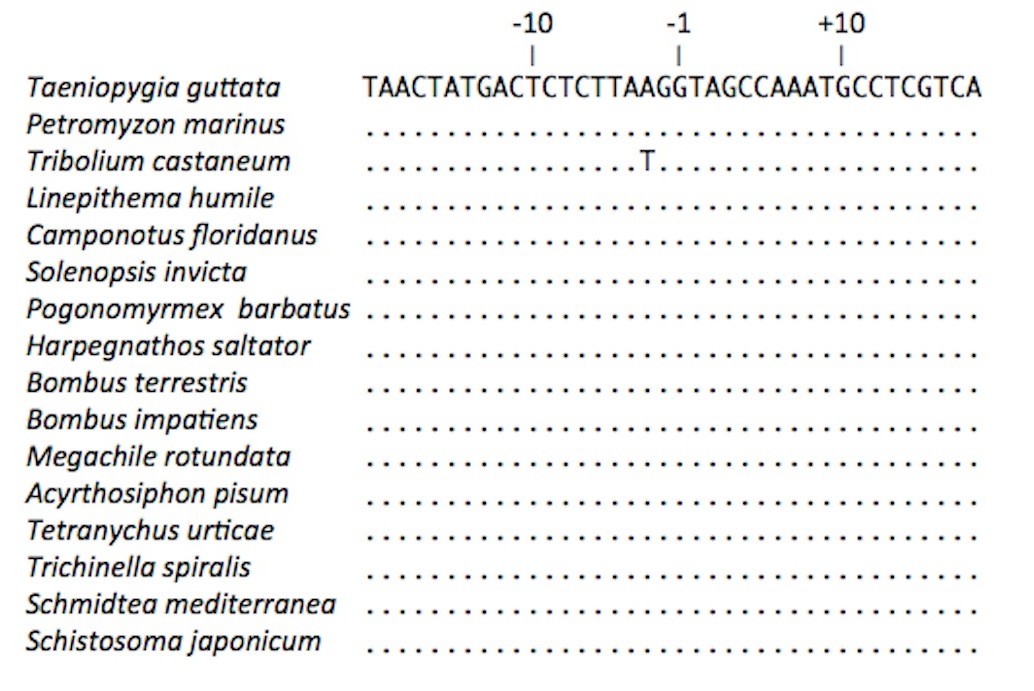


**Suppl. Figure S1** R2 insertion sites in the 28S rRNA of species where new elements has been identified.

Supplement: Figure S1 — Insertion sites within the host species 28S rRNA of newly identifies R2 elements. Alignment of the 28S’s 20 bp upstream and 20 bp downstream the R2 insertion site. (DOC) [file pone.0057076.s001.doc]
