# Supplementary material for: Non-LTR R2 Element Evolutionary Patterns: Phylogenetic Incongruences, Rapid Radiation and the Maintenance of Multiple Lineages
Source: PLoS One. 2013 Feb 25;8(2):e57076. doi: 10.1371/journal.pone.0057076 (PMC3581529; doi:10.1371/journal.pone.0057076)
Supplement: Table S1 — List of previously identified R2 sequences used for the phylogenetic analysis. Host species names, acronyms and references for each R2 element. (DOC) [file pone.0057076.s002.doc]

**Suppl. Table S1.** List of R2 sequences used for the phylogenetic analysis

| Host species | Acronyms | Reference |
| --- | --- | --- |
| *Apis mellifera* (honeybee) | R2Amel | 1 |
| *Ambiloma americanus* (tick) | R2Aam | 2 |
| *Anurida maritima* | R2Amar | 3 |
| *Argas monolakensis* (tick) | R2Amo | 2 |
| *Boophilus micropilus* (tick) | R2Bmp | 2 |
| *Ciona intestinalis* (sea squirt) | R2Ci-A | 1 |
|  | R2Ci-B | 1 |
|  | R2Ci-C | 1 |
|  | R2Ci-D | 1 |
| *Ciona savignyi* (sea squirt) | R2Cs | 1 |
| *Danio rerio* (zebrafish) | R2Dr | 1 |
| *Drosophila ambigua* (fruit fly) | R2Damb | 4 and reference cited |
| *Drosophila yakuba* (fruit fly) | R2Dyak | 4 and reference cited |
| *Drosophila melanogaster* (fruit fly) | R2Dmel | 4 and reference cited |
| *Drosophila mercatorum* (fruit fly) | R2Dmer | 4 and reference cited |
| *Drosophila sechellia* | R2Dsec | 4 and reference cited |
| *Drosophila simulans* (fruit fly) | R2Dsim | 4 and reference cited |
| *Drosophila willistoni* (fruit fly) | R2Dwill-A | 4 and reference cited |
|  | R2Dwill-B | 4 and reference cited |
| *Eptatretus burgeri* (hagfish) | R2Eb | 1 |
| *Forficula auricularia* (earwig) | R2Fa | 3 |
| *Hasarius adansoni* (jumping spider) | R2Ha | 1 |
| *Hippodamia convergens* (ladybird beetle) | R2Hc | 5 |
| *Ixodes scapolaris* (tick) | R2Is | 2 |
| *Kalotermes flavicollis* (termite) | R2Kf-1 | 6 |
|  | R2Kf-2 | 6 |
| *Lepidururs arcticus* (tadpole shrimp) | R2La | 7 |
| *Lepidurus couesii* (tadpole shrimp) | R2Lc-A | 7 |
|  | R2Lc-B | 7 |
|  | R2Lc-C | 7 |
| *Lepidurus lubbockii* (tadpole shrimp) | R2Ll | 7 |
| *Limulus polyphemus* (horseshoe crab) | R2Lp | 3 |
| *Mauremys reevesii* (turtle) | R2Cr-A | 1 |
|  | R2Cr-B1 | 1 |
|  | R2Cr-B2 | 1 |
| *Metacrinus rotundus* (sea lily) | R2Mro | 1 |
| *Nasonia vitripennis* (jewel wasp) | R2Nvit-A | 8 and reference cited |
|  | R2Nvit-B | 8 and reference cited |
| *Nasonia longicornis* (jewel wasp) | R2Nlo | 8 and reference cited |
| *Nasonia giraulti* (jewel wasp) | R2Ngi | 8 and reference cited |
| *Nematostella vectensis* (sea anemone) | R2Nvec | 9 |
| *Oryzias latipes* (medaka fish) | R2Ol | 1 |
| *Popillia japonica* (japanese beetle) | R2Pj-A | 10 |
|  | R2Pj-B | 10 |
|  | R2Pj-C | 10 |
| *Porcellio scaber* (rough woodlouse) | R2Ps | 3 |
| *Procambarus clarckii* (crayfish) | R2Pc | 1 |
| *Reticulitermes balkanensis* (termite) | R2Rbk | 6 |
| *Reticuitermes grassei* (termite) | R2Rgr | 6 |
| *Reticulitermes lucifugus* (termite) | R2Rlu | 6 |
| *Reticulitermes urbis* (termite) | R2Rur | 6 |
| *Rhyncosciara americana* (fungus gnat) | R2Ram | 11 |
| *Sciara coprophila* (fugus gnat) | R2Sc | 10 |
| *Schistosoma mansoni* (bloodfluke) | R2Sma-A | 1 |
|  | R2Sma-B | 1 |
| *Tanichthys albonubes* (minnow) | R2Ta | 1 |
| *Tenebrio molitor* (mealworm) | R2Tm-A | 5 |
|  | R2Tm-B | 5 |
| *Triops longicaudatus* (tadpole shrimp) | R2Tl | 1 |
| *Triops cancriformis* (tadpole shrimp) | R2Tc-1 | 12 |
|  | R2Tc-2 | 7 |

**References:**

1) Kojima KK, Fujiwara H (2005) Long-term inheritance of the 28S rDNA-specific retrotransposon R2. Mol Biol Evol 22: 2157-2165.

2) Bunikis J, Barbour AG (2005) Ticks have R2 retrotransposons but not the consensus transposon target site of other arthropods. Insect Mol Biol 14: 465-474.

3) Burke WD, Malik HS, Jones JP, Eickbush TH (1999) The domain structure and retrotransposition mechanism of R2 elements are conserved throughout arthropods. Mol Biol Evol 16: 502-511.

4) Stage DE, Eickbush TH (2009) Origin of nascent lineages and the mechanisms used to prime second-strand DNA synthesis in the R1 and R2 retrotransposons of *Drosophila*. Genome Biol 10: R49

5) Burke WD, Malik HS, Lathe WC 3^rd^, Eickbush TH (1998) Are retrotransposons long-term hitchhikers? Nature 392: 141–142.

6) Ghesini S, Luchetti A, Marini M, Mantovani B (2011) The Non-LTR retrotransposon R2 in termites (Insecta, Isoptera): characterization and dynamics. J Mol Evol 72: 296-305.

7) Luchetti A, Mingazzini V, Mantovani B (2012) 28S junctions and chimeric elements of the rDNA targeting non-LTR retrotransposon R2 in crustacean living fossils (Branchiopoda, Notostraca). Genomics 100: 51–56.

8) Stage DE, Eickbush TH (2010) Maintenance of multiple lineages of R1 and R2 retrotransposable elements in the ribosomal RNA gene loci of *Nasonia*. Insect Mol Biol 19(suppl. 1): 37-48.

9) Kojima KK, Kuma K, Toh H, Fujiwara H (2006) Identification of rDNA-specific non-LTR retrotransposons in Cnidaria. Mol Biol Evol 23: 1984-1993.

10) Burke WD, Eickbush DG,. Xiong Y, Jakubczak J, Eickbush TH (1993) Sequence relationship of retrotransposable elements *Rl* and *R2* within and between divergent insect species. Mol Biol Evol 10: 163-185.

11) Rezende-Teixeira P, Siviero F, da Costa Rosa M, Machado-Santelli GM (2009) The R2 mobile element of *Rhyncosciara* *americana*: molecular, cytological and dynamic aspects. Chromosome Res 17: 455-467.

12) Mingazzini V, Luchetti A, Mantovani B (2011) R2 dynamics in *Triops cancriformis* (Bosc, 1801) (Crustacea, Branchiopoda, Notostraca): turnover rate and 28S concerted evolution. Heredity 106: 567-575.
